# Recovery of effective HIV-specific CD4^+^ T-cell activity following antiretroviral therapy in paediatric infection requires sustained suppression of viraemia

**DOI:** 10.1097/QAD.0000000000001844

**Published:** 2018-07-02

**Authors:** Emily Adland, Luisa Mori, Leana Laker, Anna Csala, Maximilian Muenchhoff, Alice Swordy, Masa Mori, Philippa Matthews, Gareth Tudor-Williams, Pieter Jooste, Philip Goulder

**Affiliations:** a Department of Paediatrics, University of Oxford, Oxford, UK; b Department of Paediatrics, Kimberley Hospital, Northern Cape, South Africa; c Nuffield Department of Medicine, University of Oxford, Oxford; d Department of Paediatrics, Imperial College, London, UK.

**Keywords:** adolescence, antiretroviral therapy, CD4^+^ T cell, HIV, immune reconstitution, paediatrics, T-cell polyfunctionality

## Abstract

**Background:**

The success of increasing access to antiretroviral therapy (ART) in paediatric HIV infection prompts the question of the potential for eradication of HIV infection in this age group. ‘Shock-and-kill’ HIV cure approaches, currently in development, may depend upon an effective antiviral T-cell response to eradicate virus-infected cells.

**Method:**

We here investigate the ability of HIV-infected children receiving ART from early childhood (median 24 months’ age) to generate effective HIV-specific CD4^+^ and CD8^+^ T-cell immune responses that would facilitate future immune-based cure therapies.

**Results:**

Initial analysis of ART-naive HIV-infected children demonstrated that maintenance of normal-for-age absolute CD4^+^ T-cell counts was strongly linked to high IL-2 production and polyfunctional HIV-specific CD4^+^ T-cell responses (*P* < 0.0001 in each case). Low viral load was, similarly, strongly associated with markedly low IFN-γ and high IL-2 HIV-specific CD4^+^ T-cell responses (*P* < 0.0001). In children receiving ART, establishment of this immune profile (high IL-2 and low IFN-γ HIV-specific T-cell production) was strongly related to the duration of viraemic suppression. Failure to suppress viraemia on ART, and even the successful suppression of viraemia interrupted by the occurrence of transient viraemia of more than 1000 HIV copies/ml, was associated with an immune profile of high IFN-γ and low IL-2 HIV-specific T-cell responses and low polyfunctionality.

**Conclusion:**

These data are consistent with recovery of functional CD4^+^ T-cell responses in ART-treated children, in contrast to relative lack of CD4^+^ T-cell function recovery described in ART-treated adults. However, the challenges of achieving long-term suppression of viraemia in ART-treated children through adolescence remain daunting.

## Introduction

The challenges posed currently by paediatric HIV infection differ increasingly from those presented in the earlier decades of the global epidemic. In sub-Saharan Africa, where 90% of HIV infected children live, strategies to prevent mother-to-child transmission (MTCT) have reduced MTCT rates to 1–2% from ∼40% in the preantiretroviral therapy (ART) era [1]. The numbers of new paediatric infections are therefore decreasing (http://www.unaids.org/en/resources/fact-sheet). Furthermore, increased use of ART has made an especially dramatic impact in paediatric infection, as without ART, approximately 50% of HIV-infected children have died by 2 years of age [2]. These successes bring the new prospect of a lifetime of ART dependence for children, the cost and potential toxicity of long-term ART taken throughout childhood, the reality of poor ART adherence often accompanying adolescence [3–7]. Resources that have been successfully invested into ART initiation now also are required to maintain uninterrupted maintenance of treatment.

These factors prompt the need to consider alternative options for HIV-infected children other than the prospect of a lifetime on ART. Reports of posttreatment remission of HIV infection in children receiving ART early in life [8,9] suggest that in children there may be unique possibilities for immune-based therapies [2,10–12], as a result of the impact of immune ontogeny [13]. A number of studies have demonstrated more rapid immune reconstitution in ART-treated children compared with adults [14–18] and in part this has been related to increased thymic output [19]. However, many of these studies have focused principally on recovery of CD4^+^ T-cell numbers [20,21] as opposed to functional reconstitution.

Previous studies that have investigated immune reconstitution of T-cell function in children have, with notable exceptions [22], demonstrated clear-cut functional differences between infected adults and children on ART. For example, children aged more than 5 years achieving suppression of viraemia on ART showed strong Gag-specific CD4^+^ T-cell proliferative capacity, whereas chronically infected adults who also achieved suppression of viraemia on ART typically showed no Gag-specific proliferative response [23]. CD4^+^ proliferation requires suppression of viraemia, whereas IFN-γ production does not [24]. In perinatally-infected children aged 10–11 years with suppressed viraemia, the dominant CD4^+^ Gag-specific response was IL-2 production, whereas in children with uncontrolled viraemia, IFN-γ CD4^+^ T-cell activity predominated [25]. These findings would be consistent with a more differentiated CD4^+^ T-cell compartment in the setting of persistent viraemia, with effector memory responses characterized by IFN-γ production, limited proliferative activity and high expression of T-cell exhaustion markers [26,27]. In contrast, in the absence of viraemia, these findings suggest a less differentiated CD4^+^ T-cell compartment, comprising a greater proportion of naive and central memory CD4^+^ T cells, functionally designed for IL-2 production and high proliferative capacity, with relatively low expression of exhaustion markers.

Together these studies indicate, in ART-treated children aged more than 5 years with suppressed viraemia, a strong recovery of absolute CD4^+^ cell count, and CD4^+^ T-cell function results. However, there are few data describing reconstitution of CD4^+^ T-cell function in HIV-infected children in whom ART is initiated prior to 5 years of age, when the majority of HIV-infected children now initiate ART. We here undertook a study of two distinct groups of HIV-infected children to assess the recovery of CD4^+^ function in children initiating ART at a median of 2 years of age. We first defined in a group of 58 ART-naive children the HIV-specific CD4^+^ responses that were associated with maintenance of normal CD4^+^ cell counts for uninfected age-matched children. We then assessed immune function in 84 ART-treated children, categorized according to duration of ART-mediated suppression of viraemia.

## Methods

### Patients and samples

We studied functional patterns of HIV-specific T-cell responses in of 58 ART-naive children and 84 ART-treated children with chronic HIV-1 C-clade infection from Kimberley, South Africa. The 58 ART-naive children, aged 115 months (mean; interquartile range (IQR) 91–150 months), had absolute CD4^+^ cell counts of 850 cells/μl (mean; IQR 512–1050); CD4^+^% 26.5% (mean; IQR 21.9–30.4%) and viral load 32 909 (median; IQR 9947–153 674) had not met 2010 WHO clinical or CD4^+^ criteria for ART initiation (being: age < 1 year; or age 1–4 years: CD4^+^ < 750 or CD4^+^% < 25%; age > 5 years: CD4^+^ < 350; or WHO clinical disease stage 3 or 4).

The ART-treated children (*n* = 84) were aged 80 months (IQR 53–103), all of whom had met the CD4^+^ criteria to commence ART described above. CD4^+^ cell counts and viral loads at ART initiation and at the time of evaluations here are shown in Suppl Table 1. ART-treated children were grouped as follows: ‘Viraemic nonsuppressors’ did not achieve suppression of viraemia (<50 HIV copies/ml). ‘Aviraemic suppressors’ maintained viraemic suppression, other than for isolated ‘blips’ (viral load of <1000 copies/ml followed by a viral load of <50 copies/ml at the following timepoint). In ‘Transient Suppressors’, viral loads of less than 50 copies/ml were interrupted by spikes of viraemia (>1000 HIV copies/ml). Viraemic spikes in this group ranged from 1180 to 450 000 copies/ml (median 159 644 copies/ml), and arose a mean of eight times (range 4–15) over 6.5 years of follow-up on ART. These spikes were likely due to nonadherence as suppression of viraemia followed on the same medication.

Viral loads were determined using the BioMérieux NucliSens Version 2.0 Easy Q/Easy Mag (NucliSens v 2.0; Boston, Massachusetts, USA) assay prior to 2010 and using the COBAS AmpliPrep/COBAS TaqMan HIV-1 Test version 2.0 by Roche (CAP/CTM v 2.0; Roche Molecular Diagnostics, Pleasanton, California, USA) subsequent to 2010. CD4^+^ T-cell counts were measured by flow cytometry.

Informed consent was provided for participation of the individuals in the study. Ethics approval was given by the University of the Free State Ethics Committee, Bloemfontein, and the Oxford Research Ethics Committee.

### Whole blood intracellular cytokine assay

The whole blood intra-cellular staining assay was designed to quantitate the frequency of specific CD4^+^ and CD8^+^ T cells in small volumes of whole blood from infants in developing countries [28]. For each antigen investigated, 1-ml sodium heparinised whole blood was incubated within 2 h after collection in the presence of anti-CD28 (BD Biosciences, Oxford, UK) and anti-CD49d (BD Bioscience). Antigens used included Gag peptide pool (2 μg/ml), Pol peptide pool (2 μg/ml), Nef peptide pool (2 μg/ml), CMV lysate (NIH AIDS Reagent Programme, 5 mg/ml) and noninfected cell extract (NIH AIDS Reagent Programme, 5 mg/ml). After 5 h incubation at 37 °C, 5-μg/ml brefeldin A (Sigma-Aldrich, Gillingham, Dorset, UK) was added and incubated for a further 5 h at 37 °C. A unit of 2 mmol/l EDTA was added for 15 min at room temperature (RT). A unit of 9 ml of 1×fluorescence-activated cell sorting Lysing solution (BD Biosciences) was added and incubated for 10 min at RT. The cells were washed with PBS before being cryopreserved at −80 °C. Cell surface and intracellular staining following thawing of cryopreserved cells were performed as previously described [28]. The stimulation and intracellular staining for cytotoxic function was performed in a similar fashion on cryopreserved peripheral blood mononuclear cells (PBMC) that had been thawed. PBMC were stimulated for 6 h with 18-mer overlapping peptide pools spanning HIV clade C Gag at a concentration of 2 μg/ml.

### Surface staining for immune activation and exhaustion markers

PBMC's were thawed in R20 medium, rested for 1 h at 37 °C, 5% CO_2_, and stained with surface markers CD3 (Pacific Orange; Invitrogen, Thermo Fisher Diagnostics, Hemel Hempstead, Hertfordshire, UK), CD4^+^ (Qdot 605; Invitrogen), CD8^+^ (Pacific Blue, BD Biosciences), Live/Dead vivid marker (A750; Invitrogen), HLA-DR FITC (BD Biosciences), CD38 PECy7 (BD Biosciences), programmed death 1 (PD-1) APC (eBioscience, Thermo Fisher Diagnostics), Tim3 (PECy7; Biolegend, London, UK) and CD95 (APC; Biolegend), for 30 min at RT in the dark. Cells were then washed twice in PBS and fixed in 2% paraformaldehyde. Samples were acquired on an LSR II (Becton Dickinson, Wokingham, Berkshire, UK) flow cytometer within 12 h of staining and analysed using FlowJo version 8.8.6; FlowJo LLC, Ashland, Oregon, USA).

### Statistical analysis

Comparisons of clinical parameters in groups of children were performed using the Kruskal–Wallis test. Correlations were analysed using the Spearman Correlation test.

## Results

### High IL-2 production and polyfunctionality in HIV-specific CD4^+^ and CD8^+^ T-cell activity in nonprogressing paediatric infection

**Fig. 1:**
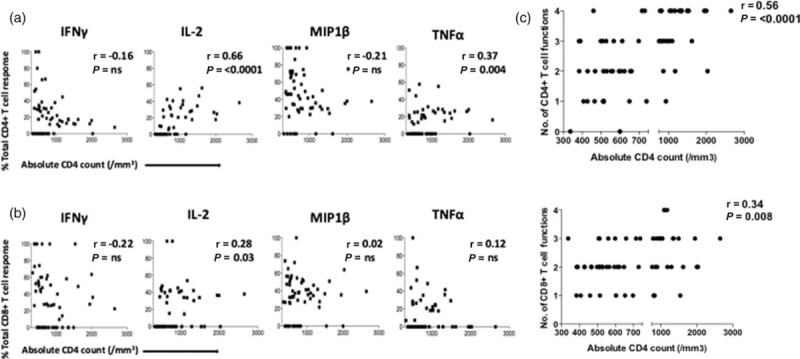
Increasing absolute CD4^+^ cell count is associated with an increase in IL-2 production in CD4^+^ and CD8^+^ T cells in HIV infected antiretroviral therapy naive children.

To define the functional profile of CD4^+^ and CD8^+^ T-cells in ART-naive children who maintain absolute CD4^+^ cell counts that are normal-for-age for HIV-uninfected children [29,30], we first analysed a mixed cohort of HIV-infected infected, ART-naive children whose absolute CD4^+^ cell counts ranged from 383 to 2650 cells/μl (Suppl Table 2). These children had a mean age of 9.6 years, CD4^+^ cell count of 850 cells/μl (IQR 512–1050) and median viral load 32 909 (IQR 9947–153 674). We first evaluated the CD4^+^ and CD8^+^ T cells in these individuals on the basis of their ability to secrete IFN-γ, IL-2, MIP1β and TNF-α after stimulation with an HIV-1 Gag peptide pool (Fig. 1).

Percentage of contribution of the indicated functional response towards the total (a) CD4^+^ T-cell and (b) CD8^+^ T-cell responses to HIV Gag peptide pool stimulation. Spearman correlations shown. (c) Polyfunctional T cells are associated with increasing absolute CD4^+^ cell count. *Y*-axis indicates the number of functional responses (IFN-γ, IL-2, MIP1β and TNF-α) made by each subject in response to HIV gag stimulation in (c1) CD4^+^ T cells and (c2) CD8^+^ T cells. *X*-axis indicates absolute CD4^+^ cell count. Spearman correlations shown.

With respect to HIV-specific CD4^+^ T-cell responses (Fig. 1a), the relative contributions to the total Gag-specific CD4^+^ T-cell response of IL-2 and TNF-α production were strongly correlated with increasing absolute CD4^+^ cell count (*r* = 0.66, *P* ≤ 0.0001; and *r* = 0.37, *P* = 0.004, respectively), whereas that of IFN-γ and MIP1β was not significantly correlated with absolute CD4^+^ cell count. For CD8^+^ T cells (Fig. 1b), the relative contribution to the total Gag-specific CD8^+^ T-cell response of IFN-γ, MIP1β and TNF-α responses was not significantly correlated with absolute CD4^+^ cell count, whereas that of IL-2 production to the total CD8^+^ T-cell response increased somewhat in association with absolute CD4^+^ cell count (*r* = 0.28, *P* = 0.028). IL-2 production by CD8^+^ and CD4^+^ T cells in response to Gag was the cytokine most strongly correlated with increasing absolute CD4^+^ cell count (*r* = 0.72, *P* < 0.0001; and *r* = 0.37, *P* = 0.003, respectively; Suppl Fig. 3), whereas IFN-γ production by CD8^+^ and CD4^+^ T cells was unrelated to absolute CD4^+^ cell count. Similar results were observed when Pol or Nef pools were used to stimulate CD4^+^ or CD8^+^ T cells in these children (Suppl Table 3).

We next evaluated T-cell polyfunctionality in the ART-naive children as polyfunctionality has previously been associated with viral control in adults [31–33]. As expected, increasing polyfunctionality in both CD8^+^ T cells and CD4^+^ T cells responding to Gag was associated with increasing absolute CD4^+^ T-cell count, especially so in the case of CD4^+^ T-cell responses (*r* = 0.56, *P* ≤ 0.0001; Fig. 1c). The polyfunctional HIV-specific T cells expressed predominantly the terminally differentiated effector memory phenotype CD45RA^+^/CCR7^−^. Similar findings were observed in response to the HIV-Pol and Nef peptide pools also tested (Suppl Table 3).

### Viral load is associated with high IFN-γ, low IL-2, TNF-α and MIPβ HIV-specific CD4^+^ T-cell responses

**Fig. 2:**
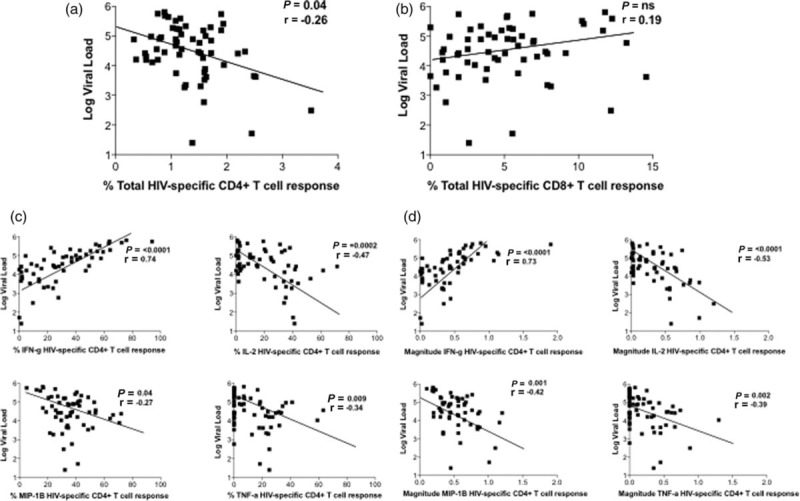
Viraemia is positively related to CD4^+^ IFN-γ and inversely related to CD4^+^ IL-2, TNF-α and MIPβ responses to HIV Gag peptide pool stimulation.

We next assessed the relationship between viral load and CD4^+^ and CD8^+^ T-cell function. The total Gag-specific CD4^+^ T-cell response was significantly related to decreasing viral load (*r* = −0.26, *P* = 0.04, Fig. 2a), whereas the total Gag-specific CD8^+^ T-cell response was not (*r* = 0.19, *P* = ns, Fig. 2b). Similarly, CD8^+^ responses stratified by IFN-γ, IL-2, MIP1β and TNF-α function were unrelated to viral load (data not shown). Among the Gag-specific CD4^+^ T-cell responses, the percentage of contribution of IFN-γ production by CD4^+^ T cells was associated with increasing viral load (*r* = 0.73, *P* ≤ 0.0001, Fig. 2c) and IL-2, TNF-α and MIP1β responses with decreasing viral load (*r* = −0.53, *P* ≤ 0.0001; *r* = −0.42, *P* = 0.001; *r* = −0.39, *P* = 0.002, respectively, Fig. 2c). The same result was observed when quantifying the magnitude of the indicated functional response, IFN-g production being positively associated with viral load (*r* = 0.73, *P* ≤ 0.0001) and IL-2, TNF-α and MIP1B responses being associated with decreasing viral load (*r* = −0.53, *P* ≤ 0.0001; *r* = −0.42, *P* = 0.001; *r* = −0.39, *P* = 0.002, respectively, Fig. 2d) Similar relationships were observed in response to stimulation of CD4^+^ and CD8^+^ T cells with HIV-1 Pol and Nef peptide pools (Suppl Table 3). Thus, the functionality of the HIV-specific CD4^+^ T-cell response, but not the CD8^+^ T-cell response, is significantly associated with viral load among these ART-naive HIV-infected children.

Viral load is (a) inversely correlated with the total percentage of Gag-specific CD4^+^ T-cell response (*P* = 0.04, *r* = −0.26); (b) not associated with the total percentage of Gag-specific CD8^+^ T-cell response (*P* = ns, *r* = 0.19); (c) positively correlated with percentage of CD4^+^ T-cell IFN-γ production (*P* ≤ 0.0001, *r* = 0.73); and inversely correlated to percentage of CD4^+^ T-cell IL-2 production (*P* ≤ 0.0001, *r* = −0.53); MIP1β production (*P* = 0.001, *r* = −0.42); and TNF-α production (*P* = 0.002, *r* = −0.39); (d) positively correlated with magnitude of CD4^+^ T-cell IFN-γ production (*P* ≤ 0.0001, *r* = 0.73); and inversely correlated to magnitude of CD4^+^ T-cell IL-2 production (*P* ≤ 0.0001, *r* = −0.53); MIP1β production (*P* = 0.001, *r* = −0.42) and TNF-α production (*P* = 0.002, *r* = −0.39); *n* = 58. Spearman correlations shown.

### Increased duration of viral suppression in antiretroviral therapy-treated children is associated with increased IL-2 and decreased IFN-γ production in CD8^+^ and CD4^+^ T cells

To determine whether ART-treated children can generate the same functional HIV-specific CD8^+^ and CD4^+^ T-cell activity as in ART-naive paediatric nonprogressors, we recruited 84 HIV-positive, ART-treated children. These were categorized into three groups: children who achieved control of viraemia to less than 50 copies/ml and maintained viral load thereafter at less than 400 copies/ml (‘Aviraemic suppressors’, *n* = 55); children in whom suppression of viraemia was periodically interrupted by transient viraemia (>1000 HIV copies/ml) (‘Transient suppressors’) (*n* = 9); and children who failed to suppress viraemia to less than 50 copies/ml (‘Viraemic nonsuppressors’, *n* = 20) (Suppl Table 1). The age of children at ART initiation did not differ between the three groups (23–24 months), nor did viral load, absolute CD4^+^ cell count or CD4^+^%.

**Fig. 3:**
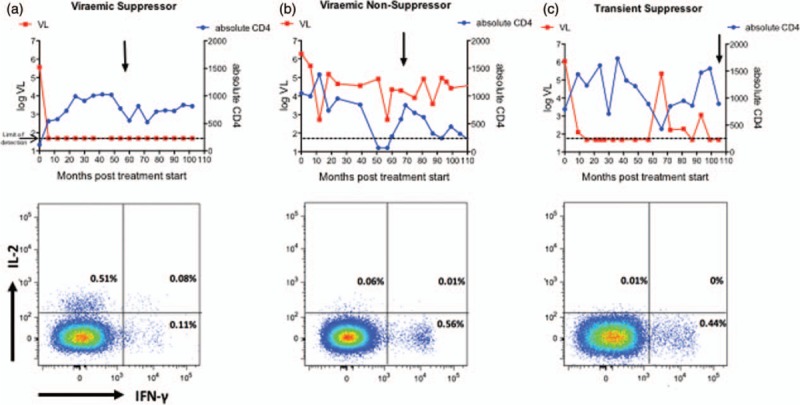
Representative clinical data for an aviraemic suppressor, a viraemic nonsuppressor and a transient suppressor.

**Fig. 4:**
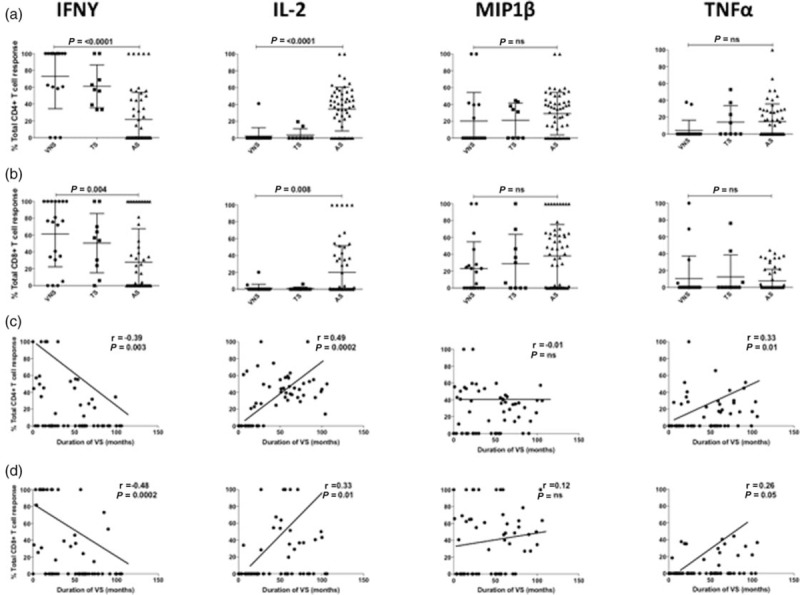
An increase in the duration of viraemic suppression is associated with a decrease in IFN-g production and an increase in IL2 production in CD4^+^ and CD8^+^ T cells in HIV infected antiretroviral therapy treated children.

In children who maintained suppression of viraemia on ART, the relative contribution of IFN-γ production to the total Gag-specific CD4^+^ and CD8^+^ T-cell response was significantly lower, whereas that of IL-2 substantially higher, than in children who failed to maintain suppression of viraemia (Figs. 3 and 4a and b). Of note, among the ‘transient suppressor’ children in whom viraemic episodes interrupted successful periods of aviraemia, HIV-specific CD4^+^ and CD8^+^ T-cell responses were indistinguishable from those of children who failed to achieve undetectable viral loads on ART (Figs. 3 and 4a and b). Among all the children studied, IL-2 production increased and IFN-γ production decreased in association with increasing duration of viral suppression (Fig. 4c and d). With respect to polyfunctionality, increasing polyfunctionality was strongly associated with duration of viraemic suppression in the case of CD4^+^ T cells (*r* = 0.55, *P* < 0.0001) but not in the case of CD8^+^ T cells (*r* = 0.26, *P* = ns, Suppl Fig. 1). Similar observations were made following stimulation of CD4^+^ and CD8^+^ T cells with Pol and Nef peptide pools (Suppl Table 3).

Absolute CD4^+^ cell count shown in blue and viral load shown in red. Vertical black arrows indicate the timepoint of the assay. In the bottom panel, CD4^+^ T-cell plots are shown for these representative subjects after stimulation with the HIV-Gag peptide pool with IFN-γ production on the *x*-axis and IL-2 production on the *y*-axis.

Percentage of contribution of the indicated functional response towards the total (a) CD4^+^ T-cell and (b) CD8^+^ T-cell responses to HIV Gag peptide pool stimulation. Viraemic nonsuppressors, *n* = 20, transient suppressors, *n* = 9; aviraemic suppressors, *n* = 55. *P* values obtained from analysis of variance. Percentage of contribution of the indicated functional response towards the total (c) CD4^+^ T-cell and (d) CD8^+^ T-cell responses to HIV Gag peptide pool stimulation in aviraemic suppressors *n* = 55. *X*-axis denotes duration of viraemic suppression in months. Spearman correlations shown.

In summary, increasing duration of viraemic suppression in ART-treated HIV-infected children is associated with restoration of the profile of CD4^+^ T-cell function that is observed in ART-naive HIV-infected children who maintain normal-for-age absolute CD4^+^ cell counts, namely decreased IFN-γ production, increased IL-2 production and increased polyfunctionality in response to stimulation by pools of peptides spanning the HIV proteins Gag, Pol and Nef. This same broad phenotype was expressed in CD8^+^ T cells in ART-treated children maintaining viraemic suppression and in ART-naive children maintaining normal-for-age absolute CD4^+^ cell counts; however, the associations with decreased IFN-γ production, increased IL-2 production and increased polyfunctionality were consistently weaker for CD8^+^ T cells than for CD4^+^ T cells.

### A similar HIV-specific cytolytic CD4^+^ phenotype is observed in antiretroviral therapy-naive children with low viraemia and in antiretroviral therapy-treated aviraemic suppressors

**Fig. 5:**
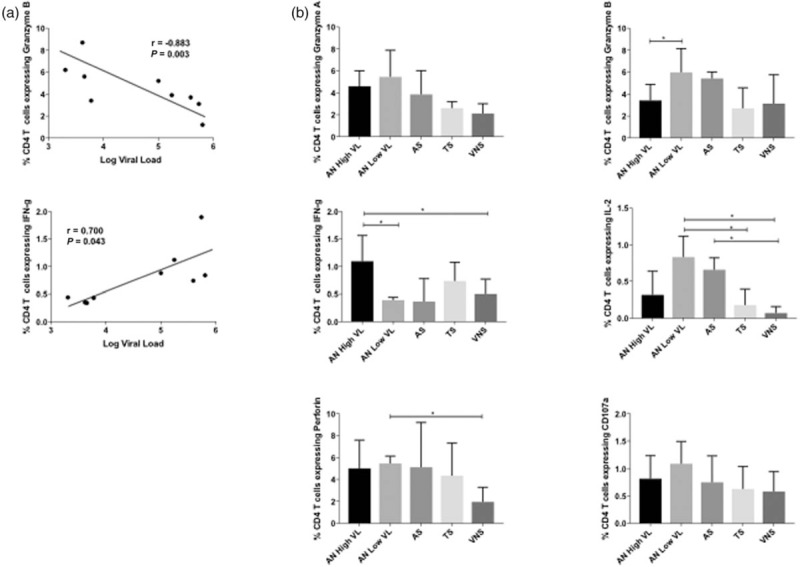
Antiretroviral therapy naïve children who maintain low viral loads exhibit an HIV-specific cytolytic CD4^+^ phenotype similar to aviraemic suppressors.

To further investigate the quality of HIV-specific CD4^+^ T-cell responses in ART-treated children we examined in a subset of children the cytolytic phenotype of the HIV-specific CD4^+^ T-cell response to HIV Gag stimulation, as assessed by the expression of granzyme A, granzyme B, perforin and CD107a. In the ART-naive children, as before we observed a strong positive correlation between IFN-γ production and viral load, and inverse correlations with markers of cytolytic activity, most significantly between Granzyme B production and viral load (Fig. 5a). Among the ART-treated children, again we observed strong similarities and no significant differences between the responses made by children maintaining viraemia on ART and those made in ART-naive children with low viral loads (<10 000 HIV RNA copies/ml) (Fig. 5b).

(a) Viral load in antiretroviral therapy naïve children is inversely associated with CD4^+^ T-cell Granzyme B production (*P* = 0.03, *r* = −0.883) (a1), and positively associated with CD4^+^ T-cell IFN-g production (*P* = −0.043, *r* = 0.700) (a2), *n* = 9. Spearman correlations shown. (b) percentage of CD4^+^ T cells expressing the relevant function as indicated after HIV-gag peptide pool stimulation in ART-naive children with ‘high’ viral load, high defined as more than 100 000 copies/ml *n* = 5; ART-naive children with ‘low’ viral load, low defined as less than 10 000 copies/ml *n* = 4; aviraemic suppressors *n* = 4; transient suppressors *n* = 4; and viraemic nonsuppressors *n* = 5. Mann–Whitney *t* tests shown ∗ indicates *P* ≤ 0.05.

### Enhanced T-cell functionality is associated with low immune activation and low exhaustion

To assess the degree of residual immune activation in ART-treated children, we first determined HLA-DR/CD38 expression in PBMC from these study individuals. Reduced immune activation (HLA/DR/CD38 expression) on both CD8^+^ and CD4^+^ T cells was associated with increasing duration of viraemic control (*P* < 0.0001; and *P* < 0.0001, respectively, Suppl Fig. 2). We next examined the expression of PD-1 and T-cell immunoglobulin mucin-3 (Tim-3), cell surface negative regulators of T cells that are upregulated in the context of immune activation and markers of immune exhaustion. Similar to immune activation, PD-1 and Tim-3 expression on both CD8^+^ and CD4^+^ T cells were both reduced in association with increasing ART duration (*P* < 0.0001; and *P* < 0.0001, respectively, Suppl Fig. 2B and C). Finally, we examined CD95 expression in these ART-treated children, the CD95/APO-1/Fas receptor/ligand system, being critically involved in induction of apoptosis in mature T cells [34]. As anticipated, we observed a significant decrease in the percentage of cells expressing CD95 in association with increasing duration of viraemic control in both the CD8^+^ and CD4^+^ T-cell subsets (*P* ≤ 0.0001 and 0.0001, respectively, Suppl Fig. 2D).

## Discussion

These analyses were designed to determine the impact of ART in restoring HIV-specific T-cell function in HIV-infected children in whom ART was initiated in the first 3 years of life. We first demonstrated that maintenance of with normal-for age CD4^+^ cell counts among ART-naïve children was associated with decreased expression of IFN-γ, increased expression of IL-2 and increased polyfunctionality of CD4^+^ and CD8^+^ T cells in response to stimulation with pools of HIV-1 Gag, Pol and Nef peptides. CD4^+^ T-cell functions were consistently more strongly associated than CD8^+^ T-cell functions with absolute CD4^+^ cell count. Similarly, immune-mediated control of viraemia in ART-naive children was strongly associated with this same immune profile. Among 84 HIV-infected children initiating ART at mean age of 24 months, three groups were distinguished: those achieving and sustaining suppression of viraemia on ART; those achieving suppression of suppression of viraemia, sustained apart from periodic episodes of viraemia (>1000 HIV copies/ml); and those not achieving sustained suppression of viraemia. Only in the group of ART-treated children who maintained viral suppression was the immune profile characteristic of disease nonprogression in the ART-naive children observed.

It is important to note that the three treatment groups did not differ by age, viral load, CD4^+^% or absolute CD4^+^ cell count prior to ART initiation (Suppl Table 1). Also, although duration of ART and age at the time the current analysis was undertaken did differ between the three groups, these did not explain the differences in immune function observed, as the children experiencing transient viraemia were older, and were on ART for longer, than the children who maintained viral suppression; who, in turn, were older and had been treated for longer than children who did not achieve viral suppression. Also, although clearly there were differences in viral load between the three ART-treated groups of children, the absolute CD4^+^ cell counts were maintained in all three groups at levels that were normal-for-age in HIV-uninfected children [29,30].

These findings are highly consistent with previous studies [14–16,18,35] showing recovery of CD4^+^ T-cell function in children who achieve aviraemia on ART. This contrasts with the absence of an equivalent recovery in CD4^+^ T-cell function among ART-treated adults [23,36]. However, it is evident that immune recovery even in children requires sustained viraemic suppression. The immune profile of high IL-2 and low IFN-γ production in response to stimulation by HIV peptide pools, is typically observed only in ART-treated children who have maintained suppression of viraemia for 5 years or more. Furthermore, the occurrence of periodic viral blips, defined here as viral loads of 400–1000 HIV copies/ml, appears to undo that immune recovery to the extent that immune function in children exhibiting periodic viral blips does not differ significantly from ART-treated children who fail to achieve consistent suppression of viraemia at all.

The data presented for the ART-naive children demonstrate a strong relationship between viral load and HIV-specific CD4^+^ T-cell activity, however no significant correlation with CD8^+^ T-cell activity. This somewhat unexpected result is consistent with previous studies in ART-naive children [37,38], in contrast to a strong correlation between control of viraemia and magnitude of the HIV-specific CD4^+^ T-cell response [37,38]. What drives this relationship between the HIV-specific CD4^+^ T-cell response and control of viraemia is the combination of IL-2, MIP1β and TNF-α CD4^+^ T-cell production. These data are consistent with control of viraemia being dependent upon polyfunctional CD4^+^ T-cell activity supporting an effective CD8^+^ T-cell response characterized by low immune activation and low expression of immune checkpoint markers such as PD-1 and Tim-3. High magnitude HIV-specific CD8^+^ T-cell responses are often driven by high viraemia, in the setting of a more differentiated CD4^+^ effector memory T-cell compartment, with high immune activation, high expression of exhaustion markers and functionality limited to IFN-γ production [38–41]. These findings therefore support the concept that CD4^+^ T-cell function is central to maintenance of nonpathogenesis in paediatric infection and that CD8^+^ T-cell activity is secondary.

In relation to HIV ‘shock and kill’ cure strategies currently being developed [42], the recovery of HIV-specific CD4^+^ T-cell function in infected children receiving ART initiated in early life suggests that CD8^+^ T-cell activity in these children will be capable of contributing to eradication of the viral reservoir. However, on the basis of the data presented here, this does depend on maintained viral suppression for a period of approximately 5 years or more. Transient viraemia may rapidly undo the painstaking work of years of immune restoration.

These studies have focused on children initiating ART at median age 24 months. In the case of HIV-infected children in whom ART is initiated in the first days or weeks of life, in whom suppression of viraemia may be established very early, it is not known what HIV-specific T-cell responses may be detectable following long-term suppression of viraemia. In the Mississippi child [8], in whom ART was initiated at 30 h of age, no virus-specific T-cell responses were detectable prior to viral rebound off ART. However, most HIV-infected infants have pre-ART viral loads of ∼10^6^ copies/ml [43]; and HIV-specific CD8^+^ T-cell responses are detectable at birth in the majority of infected babies [44,45]. Thus, early ART would not typically prevent priming of HIV-specific responses by HIV. Nonetheless, it may be necessary to boost HIV-specific T-cell responses via T-cell vaccination in ART-treated children, either to augment preexisting T-cell activity, or to prime novel and potentially more effective responses and pave the way for ART discontinuation.

## Acknowledgements

### Conflicts of interest

There are no conflicts of interest.

## Supplementary Material

Supplemental Digital Content

## References

[1] Moodley P Parboosing R Moodley D Reduction in perinatal HIV infections in KwaZulu-Natal, South Africa, in the era of more effective prevention of mother to child transmission interventions (2004–2012). *J Acquir Immune Defic Syndr* 2013; 63:410–415. 23535294 10.1097/QAI.0b013e3182926931

[2] Goulder PJ Lewin SR Leitman EM Paediatric HIV infection: the potential for cure. *Nat Rev Immunol* 2016; 16:259–271. 26972723 10.1038/nri.2016.19 PMC5694689

[3] Krogstad P Patel K Karalius B Hazra R Abzug MJ Oleske J Incomplete immune reconstitution despite virologic suppression in HIV-1 infected children and adolescents. *AIDS* 2015; 29:683–693. 25849832 10.1097/QAD.0000000000000598 PMC4391276

[4] Jaspan HB Mueller AD Myer L Bekker LG Orrell C Effect of caregivers’ depression and alcohol use on child antiretroviral adherence in South Africa. *AIDS Patient Care STDS* 2011; 25:595–600. 21470047 10.1089/apc.2010.0323 PMC3183652

[5] Becker SL Dezii CM Burtcel B Kawabata H Hodder S Young HIV-infected adults are at greater risk for medication nonadherence. *MedGenMed* 2002; 4:21. 12466764

[6] Naidoo K Munsami A Archary M Adolescent antiretroviral management: Understanding the complexity of nonadherence. *S Afr Med J* 2016; 105:953. 10.7196/samj.2015.v105i11.10150 26937510

[7] Bailey H Cruz MLS Songtaweesin WN Puthanakit T Adolescents with HIV and transition to adult care in the Caribbean, Central America and South America, Eastern Europe and Asia and Pacific regions. *J Int AIDS Soc* 2017; 20:50–59. 10.7448/IAS.20.4.21475 PMC5577698 28530040

[8] Persaud D Gay H Ziemniak C Chen YH Piatak M Jr Chun TW Absence of detectable HIV-1 viremia after treatment cessation in an infant. *N Engl J Med* 2013; 369:1828–1835. 24152233 10.1056/NEJMoa1302976 PMC3954754

[9] Frange P Faye A Avettand-Fenoel V Bellaton E Descamps D Angin M HIV-1 virological remission lasting more than 12 years after interruption of early antiretroviral therapy in a perinatally infected teenager enrolled in the French ANRS EPF-CO10 paediatric cohort: a case report. *Lancet HIV* 2016; 3:e49–e54. 26762993 10.1016/S2352-3018(15)00232-5

[10] Tobin NH Aldrovandi GM Are infants unique in their ability to be ‘functionally cured’ of HIV-1?. *Curr HIV/AIDS Rep* 2014; 11:1–10. 24390641 10.1007/s11904-013-0189-1

[11] Klein N Palma P Luzuriaga K Pahwa S Nastouli E Gibb DM Early antiretroviral therapy in children perinatally infected with HIV: a unique opportunity to implement immunotherapeutic approaches to prolong viral remission. *Lancet Infect Dis* 2015; 15:1108–1114. 26187030 10.1016/S1473-3099(15)00052-3

[12] Rainwater-Lovett K Luzuriaga K Persaud D Very early combination antiretroviral therapy in infants: prospects for cure. *Curr Opin HIV AIDS* 2014; 10:4–11. 10.1097/COH.0000000000000127 PMC4351817 25402708

[13] Muenchhoff M Prendergast AJ Goulder PJ Immunity to HIV in early life. *Front Immunol* 2014; 5:391. 25161656 10.3389/fimmu.2014.00391 PMC4130105

[14] Sleasman JW Nelson RP Goodenow MM Wilfret D Hutson A Baseler M Immunoreconstitution after ritonavir therapy in children with human immunodeficiency virus infection involves multiple lymphocyte lineages. *J Pediatr* 1999; 134:597–606. 10228296 10.1016/s0022-3476(99)70247-7

[15] Gibb DM Newberry A Klein N de Rossi A Grosch-Woerner I Babiker A Immune repopulation after HAART in previously untreated HIV-1-infected children. Paediatric European Network for Treatment of AIDS (PENTA) Steering Committee. *Lancet* 2000; 355:1331–1332. 10776748 10.1016/s0140-6736(00)02117-6

[16] Walker AS Doerholt K Sharland M Gibb DM Response to highly active antiretroviral therapy varies with age: the UK and Ireland Collaborative HIV Paediatric Study. *AIDS* 2004; 18:1915–1924. 15353977 10.1097/00002030-200409240-00007

[17] Resino S Micheloud D Larru B Bellon JM Leon JA Resino R Immunological recovery and metabolic disorders in severe immunodeficiency HIV type 1-infected children on highly active antiretroviral therapy. *AIDS Res Hum Retroviruses* 2008; 24:1477–1484. 19018671 10.1089/aid.2008.0037

[18] Sabin CA Smith CJ d’Arminio Monforte A Battegay M Gabiano C Galli L Response to combination antiretroviral therapy: variation by age. *AIDS* 2008; 22:1463–1473. 18614870 10.1097/QAD.0b013e3282f88d02

[19] De Rossi A Walker AS Klein N De Forni D King D Gibb DM Increased thymic output after initiation of antiretroviral therapy in human immunodeficiency virus type 1-infected children in the Paediatric European Network for Treatment of AIDS (PENTA) 5 Trial. *J Infect Dis* 2002; 186:312–320. 12134227 10.1086/341657

[20] Sutcliffe CG van Dijk JH Bolton C Persaud D Moss WJ Effectiveness of antiretroviral therapy among HIV-infected children in sub-Saharan Africa. *Lancet Infect Dis* 2008; 8:477–489. 18652994 10.1016/S1473-3099(08)70180-4

[21] Sauvageot D Schaefer M Olson D Pujades-Rodriguez M O’Brien DP Antiretroviral therapy outcomes in resource-limited settings for HIV-infected children <5 years of age. *Pediatrics* 2010; 125:e1039–e1047. 20385636 10.1542/peds.2009-1062

[22] Vrisekoop N van Gent R de Boer AB Otto SA Borleffs JC Steingrover R Restoration of the CD4 T cell compartment after long-term highly active antiretroviral therapy without phenotypical signs of accelerated immunological aging. *J Immunol* 2008; 181:1573–1581. 18606713 10.4049/jimmunol.181.2.1573

[23] Feeney ME Draenert R Roosevelt KA Pelton SI McIntosh K Burchett SK Reconstitution of virus-specific CD4 proliferative responses in pediatric HIV-1 infection. *J Immunol* 2003; 171:6968–6975. 14662905 10.4049/jimmunol.171.12.6968

[24] Scott ZA Beaumier CM Sharkey M Stevenson M Luzuriaga K HIV-1 replication increases HIV-specific CD4+ T cell frequencies but limits proliferative capacity in chronically infected children. *J Immunol* 2003; 170:5786–5792. 12759463 10.4049/jimmunol.170.11.5786

[25] Correa R Harari A Vallelian F Resino S Munoz-Fernandez MA Pantaleo G Functional patterns of HIV-1-specific CD4 T-cell responses in children are influenced by the extent of virus suppression and exposure. *AIDS* 2007; 21:23–30. 17148964 10.1097/QAD.0b013e32801120bc

[26] Sallusto F Lenig D Forster R Lipp M Lanzavecchia A Two subsets of memory T lymphocytes with distinct homing potentials and effector functions. *Nature* 1999; 401:708–712. 10537110 10.1038/44385

[27] Day CL Kaufmann DE Kiepiela P Brown JA Moodley ES Reddy S PD-1 expression on HIV-specific T cells is associated with T-cell exhaustion and disease progression. *Nature* 2006; 443:350–354. 16921384 10.1038/nature05115

[28] Hanekom WA Hughes J Mavinkurve M Mendillo M Watkins M Gamieldien H Novel application of a whole blood intracellular cytokine detection assay to quantitate specific T-cell frequency in field studies. *J Immunol Methods* 2004; 291:185–195. 15345316 10.1016/j.jim.2004.06.010

[29] Shearer WT Rosenblatt HM Gelman RS Oyomopito R Plaeger S Stiehm ER Lymphocyte subsets in healthy children from birth through 18 years of age: the Pediatric AIDS Clinical Trials Group P1009 study. *J Allergy Clin Immunol* 2003; 112:973–980. 14610491 10.1016/j.jaci.2003.07.003

[30] Lugada ES Mermin J Kaharuza F Ulvestad E Were W Langeland N Population-based hematologic and immunologic reference values for a healthy Ugandan population. *Clin Diagn Lab Immunol* 2004; 11:29–34. 14715541 10.1128/CDLI.11.1.29-34.2004 PMC321349

[31] Betts MR Nason MC West SM De Rosa SC Migueles SA Abraham J HIV nonprogressors preferentially maintain highly functional HIV-specific CD8+ T cells. *Blood* 2006; 107:4781–4789. 16467198 10.1182/blood-2005-12-4818 PMC1895811

[32] Rehr M Cahenzli J Haas A Price DA Gostick E Huber M Emergence of polyfunctional CD8+ T cells after prolonged suppression of human immunodeficiency virus replication by antiretroviral therapy. *J Virol* 2008; 82:3391–3404. 18199637 10.1128/JVI.02383-07 PMC2268491

[33] Nemes E Bertoncelli L Lugli E Pinti M Nasi M Manzini L Cytotoxic granule release dominates gag-specific CD4+ T-cell response in different phases of HIV infection. *AIDS* 2010; 24:947–957. 20179574 10.1097/QAD.0b013e328337b144

[34] Strand S Hofmann WJ Hug H Muller M Otto G Strand D Lymphocyte apoptosis induced by CD95 (APO-1/Fas) ligand-expressing tumor cells – a mechanism of immune evasion?. *Nat Med* 1996; 2:1361–1366. 8946836 10.1038/nm1296-1361

[35] Resino S Resino R Micheloud D Gurbindo Gutierrez D Leon JA Ramos JT Long-term effects of highly active antiretroviral therapy in pretreated, vertically HIV type 1-infected children: 6 years of follow-up. *Clin Infect Dis* 2006; 42:862–869. 16477566 10.1086/500412

[36] Rosenberg ES Billingsley JM Caliendo AM Boswell SL Sax PE Kalams SA Walker BD Vigorous HIV-1-specific CD4+ T cell responses associated with control of viremia. *Science* 1997; 278:1447–1450. 9367954 10.1126/science.278.5342.1447

[37] Prendergast A Goodliffe H Clapson M Cross R Tudor-Williams G Riddell A Gag-specific CD4+ T-cell responses are associated with virological control of paediatric HIV-1 infection. *AIDS* 2011; 25:1329–1331. 21505296 10.1097/QAD.0b013e3283478575

[38] Prendergast A O’Callaghan M Menson E Hamadache D Walters S Klein N Goulder P Factors influencing T cell activation and programmed death 1 expression in HIV-infected children. *AIDS Res Hum Retroviruses* 2011; 28:465–468. 21834749 10.1089/AID.2011.0113

[39] Muenchhoff M Adland E Karimanzira O Crowther C Pace M Csala A Nonprogressing HIV-infected children share fundamental immunological features of nonpathogenic SIV infection. *Sci Transl Med* 2016; 8:358ra125. 10.1126/scitranslmed.aag1048 PMC6087524 27683550

[40] Wherry EJ Blattman JN Ahmed R Low CD8 T-cell proliferative potential and high viral load limit the effectiveness of therapeutic vaccination. *J Virol* 2005; 79:8960–8968. 15994790 10.1128/JVI.79.14.8960-8968.2005 PMC1168795

[41] Wherry EJ Kurachi M Molecular and cellular insights into T cell exhaustion. *Nat Rev Immunol* 2015; 15:486–499. 26205583 10.1038/nri3862 PMC4889009

[42] Deeks SG HIV: shock and kill. *Nature* 2012; 487:439–440. 22836995 10.1038/487439a

[43] Mphatswe W Blanckenberg N Tudor-Williams G Prendergast A Thobakgale C Mkhwanazi N High frequency of rapid immunological progression in African infants infected in the era of perinatal HIV prophylaxis. *AIDS* 2007; 21:1253–1261. 17545701 10.1097/QAD.0b013e3281a3bec2

[44] Thobakgale CF Ramduth D Reddy S Mkhwanazi N de Pierres C Moodley E Human immunodeficiency virus-specific CD8+ T-cell activity is detectable from birth in the majority of in utero-infected infants. *J Virol* 2007; 81:12775–12784. 17881456 10.1128/JVI.00624-07 PMC2169079

[45] Luzuriaga K Holmes D Hereema A Wong J Panicali DL Sullivan JL HIV-1-specific cytotoxic T lymphocyte responses in the first year of life. *J Immunol* 1995; 154:433–443. 7995957

