# Supplementary material for: Recovery of effective HIV-specific CD4+ T-cell activity following antiretroviral therapy in paediatric infection requires sustained suppression of viraemia
Source: AIDS. 2018 Jul 2;32(11):1413–22. doi: 10.1097/QAD.0000000000001844 (PMC6039399; doi:10.1097/QAD.0000000000001844)

**Suppl Table 1. Clinical parameters in the 3 groups of HIV infected, ART treated children characterised by degree of virological suppression (VS).** Aviraemic suppressors were defined as two consecutive viral loads of <50 copies/ml following ART initiation and maintaining VL <400 copies/ml. Intermittent viraemia for isolated ‘blips’ <1000 copies/ml was allowed. Viraemic non-suppressors did not achieve VS. ‘Transient suppressors’ had multiple consecutive ‘blips’ of viraemia >400 copies/ml. (ACC) absolute CD4 count; (VL) viral load. Values shown are the mean of the group and IQR in brackets, with the exception of viral load (VL) where the median is shown. p values obtained from Kruskal-Wallis test.


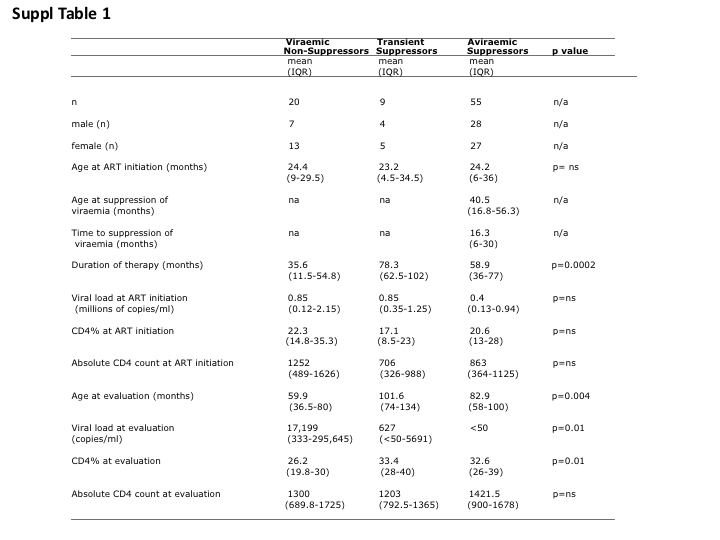


**Suppl Table 2. Clinical parameters in the 4 groups of HIV infected, ART naïve children characterised by absolute CD4 count (ACC).** Values shown are the mean of the group and IQR in brackets, with the exception of viral load (VL) where the median is shown. p values obtained from Kruskal-Wallis test.


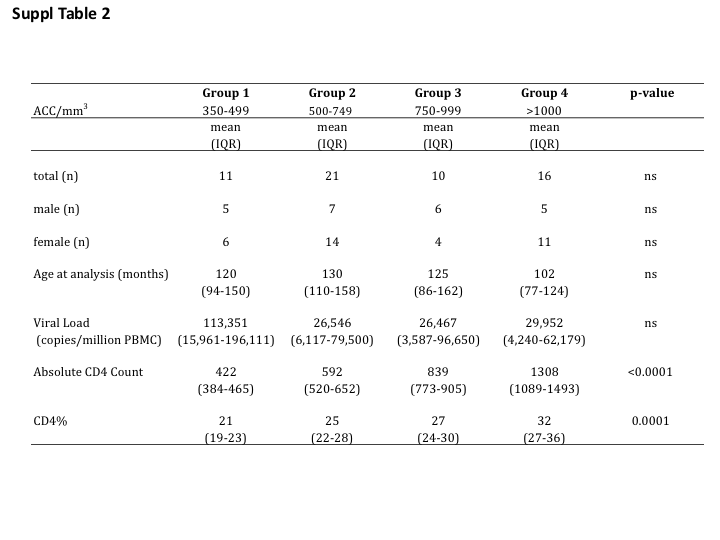


**Suppl Fig 1. Association of ART duration with restoration of CD4 but not CD8 polyfunctionality.** y axis indicates the number of functional responses (IFNγ, IL-2, MIP1β and TNFα) made by each subject in response to HIV gag stimulation in (A) CD4+ T cells and (B) CD8+ T cells. X axis indicates duration of viraemic suppression (VS) in months. Spearman correlations shown.


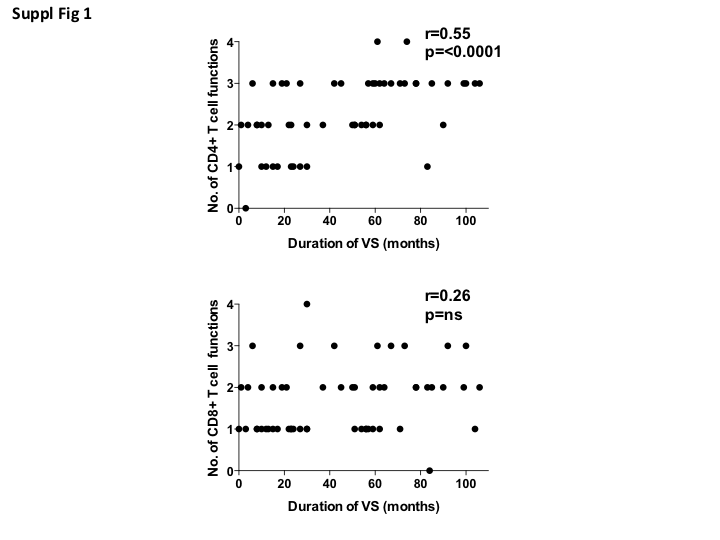


**Suppl Fig 2. Enhanced T cell functionality is associated with low immune activation and low exhaustion.** Correlations between the proportion of baseline HLA-DR+ CD38+ T-cells [A], PD-1+ T-cells [B], Tim3+ T-cells [C], and CD95+ T-cells [D], with duration of viraemic suppression in months are shown (all ART treated children n=84). Correlations for both CD8+ T-cells and CD4+ T-cells are shown. Spearman correlations shown.


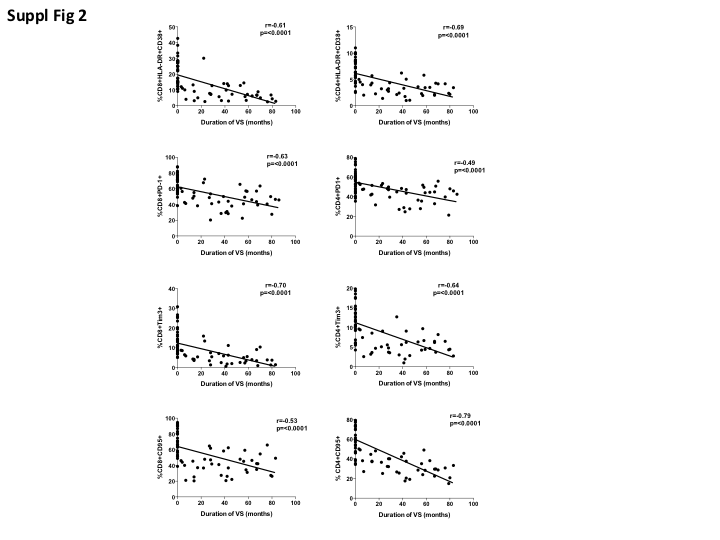


**Suppl Fig 3.** **Increasing Absolute CD4 Count (ACC) is associated with an increase in the magnitude of IL-2, MIP1β and TNFα production in both CD4+ and CD8+ T-cells in HIV infected ART naïve children**. [A] Percentage of CD4+ T cells and [B] Percentage of CD8+ T cells producing the indicated function in response to HIV Gag peptide pool stimulation. Spearman correlations shown.


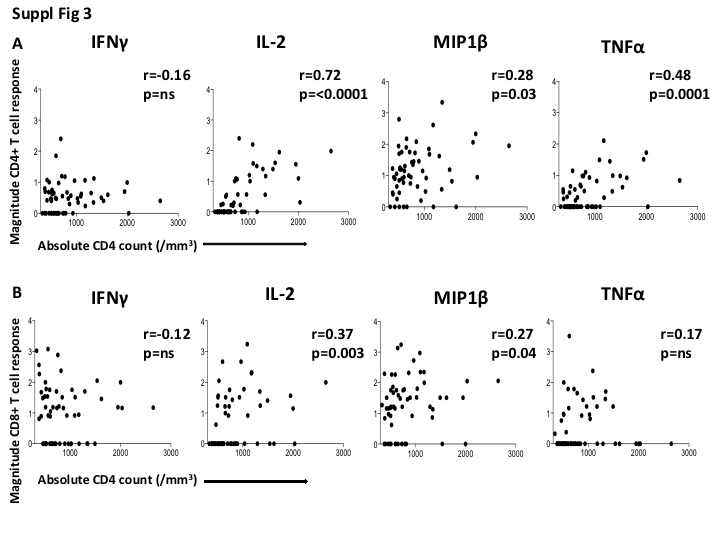


**Suppl Table 3. Functional responses of CD4+ and CD8+ T cells to HIV-gag stimulation are concordant with both HIV-pol and HIV-nef peptide pool stimulation.** % contribution of the indicated functional response toward the total CD4+ T cell and CD8+ T cell responses to HIV-pol and HIV-nef peptide pool stimulation are shown. r and p values were obtained from spearman correlations.


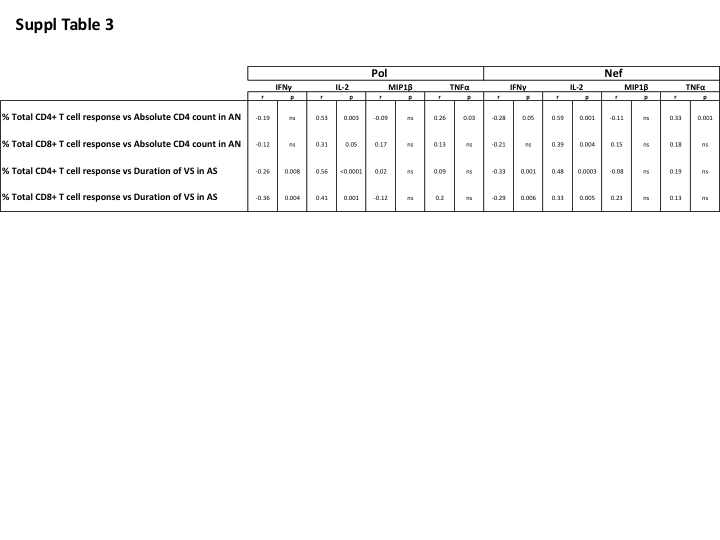

Supplement: Supplemental Digital Content [file aids-32-1413-s001.doc]
